# Climate change and women: an integrated reflection between gender and the Sustainable Development Goals

**DOI:** 10.1590/0034-7167-2024-0376

**Published:** 2025-09-01

**Authors:** Maria Valéria Chaves de Lima, Janaina Maciel de Queiroz, Kalyane Kelly Duarte de Oliveira, Antônio Rodrigues Ferreira

**Affiliations:** I Universidade Estadual do Ceará. Fortaleza, Ceará, Brazil; II Universidade Federal Rural do Semi-Árido. Mossoró, Rio Grande do Norte, Brazil; III Universidade do Estado do Rio Grande do Norte. Pau dos Ferros, Rio Grande do Norte, Brazil

**Keywords:** Sustainable Development, Climate Change, Women, Global Health Strategies, Gender Equity, Desarrollo Sostenible, Cambio Climático, Mujeres, Estrategias de Salud Globales, Equidad de Género

## Abstract

**Objectives:**

to discuss the interrelationship between climate change, gender, women’s health and the Sustainable Development Goals.

**Methods:**

a reflective article.

**Results:**

emphasis is placed on the relationship between women and climate, permeating the discussion of gender, as women stand out in the climate struggle for their roles as promoters, executors, creators and managers in numerous social contexts, whether they occupy them of their own free will or are forced to assume them due to external factors.

**Final Considerations:**

women play a vital role in natural resource management and community resilience, but they face significant challenges due to gender inequalities. To achieve a sustainable future, it is imperative that climate and development policies include and reflect women’s capabilities and needs, ensuring their full participation and leadership.

## RESUMEN

**Objetivos:**

discutir la interrelación entre cambio climático, género, salud de las mujeres y los Objetivos de Desarrollo Sostenible.

**Métodos:**

artículo tipo reflectante.

**Resultados:**

se enfatiza la relación entre las mujeres y el clima, permeando la discusión de género, ya que las mujeres destacan en la lucha climática por sus roles como promotoras, ejecutoras, creadoras y gestoras en innumerables contextos sociales, ya sea que los ocupen voluntariamente o se vean obligadas a asumirlos por factores externos.

**Consideraciones Finales:**

las mujeres desempeñan un papel vital en la gestión de los recursos naturales y la resiliencia comunitaria, pero enfrentan desafíos importantes debido a las desigualdades de género. Para lograr un futuro sostenible, es esencial que las políticas climáticas y de desarrollo incluyan e interpreten las capacidades y necesidades de las mujeres, asegurando su plena participación y liderazgo.

## INTRODUCTION

Climate change constantly affects human life. However, the phenomena resulting from these changes have different effects on men and women. It is possible to think about this perspective substantially when we observe the roles that each person occupies in society as well as the economic, social and physical resources that each person has.

There are notable sociocultural barriers involving class and gender that fall mainly on women, who have fewer mechanisms to act in the face of climate threats. Thus, the impacts of climate change disproportionately affect individuals, groups and peoples in vulnerable situations, such as older adults, indigenous people, black people, women and children, highlighting structural problems, such as poverty, education, food security, health, etc.^(1)^.

The poor and marginalized are more vulnerable to the impacts of climate change because they have few resources and, at times, depend on natural resources that are affected by these changes. In this perspective, there is a higher percentage of women living in poverty, and this level ends up making them the most affected when the climate changes drastically. It is clear that food and water and energy supply are the main means of subsistence for women in several countries, and losses in any of these components directly affect the quality of life of women^(2)^.

Moreover, the United Nations (UN) recently announced that at least 80% of people displaced due to climate change are poor girls and women who depend on agricultural activities to ensure their survival. Although data points to the correlation between gender issues and climate change, climate policies do not fully incorporate points that emphasize racial, ethnic and gender issues. Proportionally, women participate little in the development of these policies. On the other hand, women are the group most willing to change their behavior to combat the climate crisis^(3,4)^.

In this scenario, the UN has attempted to operationalize the Sustainable Development Goals (SDGs), which are characterized as a global initiative aimed at reducing inequality and poverty, improving social and economic conditions, protecting the environment and climate, and, proportionally, protecting human rights. Achieving these goals starts with the creation of targets and the observation of indicators that need to generate information and portray the realities of each of the concepts that correlate with sustainability^(5)^.

The proposal agreed upon by the UN in the 2030 Agenda to “transform the world without leaving anyone behind” represents a bold but necessary action, given the intense social inequalities present in the country and in the world and the rapid advance of the impacts resulting from climate change^(6)^.

Based on the analysis of this context, it is emphasized that the problem of climate change linked to gender issues is not new, but it permeates unresolved issues with political nuances, human rights, and workers’ health, reflecting on issues of equity and safety for women amid these changes. Thus, recognizing the difficulties experienced and the female role for sustainable development is an important step towards outlining another reality.

## OBJECTIVES

To discuss the interrelationship between climate change, gender, women’s health and the SDGs.

## METHODS

This is a theoretical reflection based on scientific studies on climate change, women and the SDGs. The reflection started from the following question: what is the relationship between climate change, gender, women’s health and the SDGs?

The literature review to support the discussion was carried out by searching for articles in the PubMed, Scientific Electronic Library Online (SciELO), Embase and Virtual Health Library (VHL) databases. The descriptors used in the strategy were “Climate Change”, “Women’s Health”, “Gender and Health” and “Sustainable Development Goals”. A total of 88 articles were found, which were initially selected by reading the title and abstract, and those selected at this stage were read in full, totaling nine articles. The study was carried out from June to September 2024.

Full texts that addressed issues related to the objective of this study were included. Duplicate articles, editorials, theses, dissertations and abstracts were excluded.

A narrative approach was used to summarize the results. The results were interpreted in light of the research question, highlighting emerging needs, gaps in evidence and implications for practice and future research. To discuss the results, references from the sample and complementary literature were used.

## RESULTS

Sustainability can be defined by three dimensions (social, economic and environmental), and can be achieved in macro (planet, country) or micro (individuals, companies, etc.) senses. Based on this, the SDGs are related to this tripod, pointing out 17 objectives, which are: 1^st^ No poverty; 2^nd^ Zero hunger; 3^rd^ Good health and well-being; 4^th^ Quality education; 5^th^ Gender equality; 6^th^ Clean water and sanitation; 7^th^ Affordable and clean energy; 8^th^ Decent work and economic growth; 9^th^ Industry, innovation and infrastructure; 10^th^ Reduced inequalities; 11^th^ Sustainable cities and communities; 12^th^ Responsible consumption and production; 13^th^ Climate action; 14^th^ Life below water; 15^th^ Life on land; 16^th^ Peace, justice and strong institutions; 17^th^ Partnerships for the goals. Therefore, the 17 goals can be understood from five spheres of relevance for the planet and humanity: peace; the planet; people; prosperity; and partnerships. The sum of these goals makes up the so-called 2030 Agenda^(7)^.

It should be noted that achieving one goal can be beneficial for achieving others. SDG 5, which concerns gender equality, for instance, directly influences the others that deal with peace and justice, food security, energy and sustainable cities. Proportionally, creating policies that allow for gender equality will give women more visibility in society and the economy, allowing them to have more support to deal with climate crises and participate in the creation of climate-related policies^(1)^.

It is worth noting that gender inequality means that women receive the lowest wages, are responsible for the extremely high rates of domestic violence, femicide, as well as sexual assault and harassment, illiteracy rates and poverty. This context, which goes against human rights, makes the need to achieve the SDG 5 targets even more urgent^(1)^.

Issues related to gender are diverse, linked to different layers of capitalism and patriarchy. It is worth noting that in many contexts, women are not introduced to learning skills, such as swimming or climbing trees, which are important for survival in climate disasters, and these types of skills are generally reserved for boys, while girls engage in more home-oriented activities. During disasters, many women also do not go to shelters for fear of being away from their children and household goods. Furthermore, women may also suffer from a lack of access to reliable information about natural disasters, which is sometimes passed on to men in public settings. In some communities, women are also often disadvantaged during disasters due to their restrictive clothing, which affects their mobility when seeking help. Women also have fewer technological resources at their disposal when searching for survival resources. This is why it is so important to address gender inequalities^(2)^.

Within this context of inequality with the female gender, there are also specificities that transcend geographic, racial, ethnic and group boundaries that require the creation of even more specific goals for these women. In countries like Brazil, *quilombola* (a resident of a “*quilombo*” in Brazil), black, indigenous, gypsy and LGBTQI+ women are among the most vulnerable and silenced^(6)^.

Women are seen as leading actors in achieving the SDGs because they are important figures in the search for a culture of peace, due to the presence of values such as solidarity and tolerance, which this group demonstrates when raising humanitarian issues. Peace is an important path to achieving sustainable development. Thus, the constant premise of ending violence that women seek serves as a step towards achieving sustainability and reducing climate change^(6)^.

Emphasizing the relationship between women and climate, also encompassing the discussion of gender, by focusing on what SDG 13 defends, women stand out in the climate fight for their roles as promoters, executors, creators and managers in numerous social contexts, whether they occupy them of their own free will or are forced to assume them due to external factors. Generally, women are the figures responsible for leading the family, organizing the home, educating their family members, acquiring and preparing water and food, and maintaining the family, in addition to their functions that are outside the context of the home^(6)^.

In order for women to be more active in achieving the SDG targets, there needs to be a convergence of public and societal actions focused on specific agenda items. In this regard, budgetary disputes between governments, authors and interests need to be overcome so that Agenda 30 can be fulfilled and women can be part of this achievement^(8)^.

## FINAL CONSIDERATIONS

Given the above, it is clear that the challenge of climate change and the pursuit of sustainable development are intrinsically linked to gender equality promotion. Women play a vital role in natural resource management and community resilience, but they face significant challenges due to gender inequalities. To achieve a sustainable future, it is essential that climate and development policies include women’s participation from their design stage. It is important that these moments of construction serve to welcome and interpret women’s capacities and needs, ensuring their full participation in the creation, continuity and leadership in spaces of governance, assistance and experience. Only through an integrated approach, which unites climate action with gender equality, will we be able to move towards a more just and sustainable world.

## References

[1] Carvalho AF, Javoni LAR, Seixas SRC. (2020). Mudanças climáticas, sustentabilidade e direitos humanos: algumas considerações sobre gênero e raça. Momentum.

[2] Silva EMV, Melo FCAB. (2020). Da teoria verde ao ecofeminismo: mulheres na África Meridional frente às mudanças climáticas. e-cadernos CES.

[3] Organização das nações Unidas (ONU) (2024). Sobre o nosso trabalho para alcançar os objetivos de desenvolvimento sustentável no Brasil.

[4] Cassab LA. (2024). Ecofeminismo, mulheres, sustentabilidade socioambiental. In: Congresso Internacional de Política Social e Serviço Social: desafios contemporâneos; Seminário Nacional de Território e Gestão de Políticas Sociais; Congresso de Direito à Cidade e Justiça Ambiental.

[5] Ferreira P, Almeida MRR. (2024). Percepção dos docentes da Universidade Federal de Itajubá sobre os objetivos de desenvolvimento sustentável. Rev GUAL.

[6] Hidalgo-Munhoz RA, Ruiz-Goulart AL, Santos RSS. (2021). Mulheres, clima e agenda 2030: narrativas verbais-imagética para não deixar ninguém para trás. Amb Educ.

[7] Traverso LD, Patias TZ, Toselli C, Silva LD. (2023). Turismo e Objetivos de Desenvolvimento Sustentável: uma análise a partir da produção nacional e das políticas públicas brasileiras. CVT.

[8] Santos GR, Nogueira D, Freitas DAF. (2023). Mulheres, água e equidade: uma agenda que faz sentido?. In: Desenvolvimento e Meio Ambiente; Seção especial - Água, Saneamento e ODS no Brasil: desafios, contradições e governança.

