# Supplementary material for: Climate change and women: an integrated reflection between gender and the Sustainable Development Goals
Source: Rev Bras Enferm. 2025 Sep 1;78(Suppl 1):e20240376. doi: 10.1590/0034-7167-2024-0376 (PMC12407853; doi:10.1590/0034-7167-2024-0376)
Supplement: Translation

# Translation: Mudanças climáticas e mulheres: uma reflexão integrada entre gênero e os Objetivos de Desenvolvimento Sustentável

**DOI:** 10.1590/0034-7167-2024-0376pt

**Authors:** Maria Valéria Chaves de Lima, Janaina Maciel de Queiroz, Kalyane Kelly Duarte de Oliveira, Antônio Rodrigues Ferreira

**Affiliations:** I Universidade Estadual do Ceará. Fortaleza, Ceará, Brasil; II Universidade Federal Rural do Semi-Árido. Mossoró, Rio Grande do Norte, Brasil; III Universidade do Estado do Rio Grande do Norte. Pau dos Ferros, Rio Grande do Norte, Brasil

**Keywords:** Sustentável, Mudança Climática, Mulheres, Estratégias de Saúde Globais, Equidade de Gênero

## Abstract

**Objetivos:**

discutir a inter-relação entre as mudanças climáticas, gênero, saúde das mulheres e os Objetivos de Desenvolvimento Sustentável.

**Métodos:**

artigo do tipo reflexivo.

**Resultados:**

enfatiza-se a relação entre mulheres e clima, perpassando a discussão de gênero, pois as mulheres se destacam na luta climática pelos seus papéis de promotoras, executoras, criadoras e gestoras em inúmeros contextos sociais, estejam elas os ocupando por vontade própria ou obrigada a assumir por fatores externos.

**Considerações Finais:**

as mulheres desempenham um papel vital na gestão dos recursos naturais e na resiliência comunitária, mas enfrentam desafios significativos devido às desigualdades de gênero. Para alcançar um futuro sustentável, é imprescindível que as políticas climáticas e de desenvolvimento incluam e interpretem as capacidades e necessidades das mulheres, garantindo sua plena participação e liderança.

## INTRODUÇÃO

As alterações climáticas afetam a vida do ser humano constantemente. Contudo, os fenômenos advindos dessas alterações possuem efeitos diferenciados em homens e mulheres. É possível pensar nesta perspectiva substancialmente quando observamos os papéis que cada um ocupa na sociedade, assim como os recursos econômicos, sociais e físicos que cada um dispõe.

Há barreiras socioculturais notórias que envolvem classe e gênero que recaem principalmente sobre o público feminino, que dispõe de menos mecanismos para agir diante de ameaças climáticas. Assim, os impactos das mudanças climáticas afetam desproporcionalmente indivíduos, grupos e povos em situações vulneráveis, como idosos, indígenas, pessoas negras, mulheres e crianças, salientando problemas estruturais, como pobreza, educação, segurança alimentar, saúde, etc.^(1)^.

Os pobres e marginalizados são mais vulneráveis aos impactos de mudanças climáticas por terem poucos recursos e, por vezes, dependerem justamente de recursos naturais que sofrem com essas alterações. Nessa perspectiva, encontra-se um maior percentual de mulheres em situação de pobreza, e esse patamar acaba por torná-las as pessoas mais prejudicadas quando o clima muda drasticamente. Evidencia-se que a alimentação e o suprimento de água e energia são os principais meios de subsistência das mulheres em diversos países, e prejuízos em qualquer um destes componentes afetam diretamente a qualidade de vida do público feminino^(2)^.

Somado a isso, recentemente, a Organização das Nações Unidas (ONU) divulgou que pelo menos 80% das pessoas deslocadas, devido às mudanças climáticas, são meninas e mulheres pobres que dependem de atividades agrícolas para garantir sua sobrevivência. Embora os dados apontem a correlação de questões de gênero a mudanças climáticas, as políticas climáticas não incorporam em sua totalidade pontos que enfatizem as problemáticas raciais, étnicas e de gênero. Proporcionalmente, as mulheres pouco participam da construção dessas políticas. Por outro lado, as mulheres são o grupo mais disposto a mudar suas condutas para combater a crise climática^(3,4)^.

Nesse cenário, a ONU tem tentado operacionalizar os Objetivos de Desenvolvimento Sustentável (ODS), que se caracterizam como uma iniciativa planetária direcionada à redução das desigualdades e pobreza, melhora das condições sociais e econômicas, proteção do meio ambiente e clima, e, proporcionalmente, proteção dos direitos humanos. O alcance desses objetivos parte da criação de metas e observação de indicadores que precisam gerar informações e retratar as realidades de cada um dos conceitos que se correlacionam com a sustentabilidade^(5)^.

A proposta pactuada pela ONU da Agenda 2030 em “transformar o mundo sem deixar ninguém para trás” representa uma ação ousada, mas necessária, diante das intensas desigualdades sociais presentes no país e no mundo e do rápido avanço dos impactos resultantes das mudanças climáticas^(6)^.

A partir da análise desse contexto, enfatiza-se que a problemática das alterações climáticas atreladas às questões de gênero não é de hoje, entretanto perpassa questões não resolvidas com nuances políticas, de direitos humanos, da própria saúde do trabalhador, refletindo nas questões de equidade e segurança do ser mulher em meio a essas alterações. Assim, reconhecer as dificuldades vivenciadas e o protagonismo feminino para o desenvolvimento sustentável é um passo importante para delinear outra realidade.

## OBJETIVOS

Discutir a inter-relação entre as mudanças climáticas, gênero, saúde das mulheres e os ODS.

## MÉTODOS

Trata-se de reflexão teórica baseada em publicações científicas sobre mudanças climáticas, as mulheres e os ODS. A reflexão partiu do seguinte questionamento: qual a relação entre mudanças climáticas, gênero, saúde da mulher e ODS?

A revisão de literatura para embasar a discursão foi realizada por meio da busca de artigos nas bases de dados PubMed, *Scientific Eletronic Library Online* (SciELO), Embase e Biblioteca Virtual em Saúde (BVS). Os descritores empregados na estratégia foram “Mudança Climática”, “Saúde da Mulher”, “Gênero e Saúde” e “Objetivos de Desenvolvimento Sustentável”. Encontraram-se 88 artigos, que foram selecionados inicialmente através da leitura do título e resumo, e os que foram selecionados nessa etapa foram lidos na íntegra, totalizando nove artigos. O estudo foi realizado no período de junho a setembro de 2024.

Foram incluídos textos completos que abordassem assuntos referentes ao objetivo deste estudo. Foram excluídos artigos duplicados, editoriais, teses, dissertações e resumos.

Para a síntese dos resultados, empregou-se uma abordagem narrativa. Interpretaram-se os resultados à luz da pergunta de pesquisa, destacando necessidades emergentes, lacunas na evidência e implicações para a prática e para futuras pesquisas. Para discutir os resultados, foram utilizadas as referências da amostra e literatura complementar.

A sustentabilidade pode ser definida por três dimensões (social, econômica e ambiental), e pode ser alcançada em sentidos macro (planeta, país) ou micro (indivíduos, empresas, etc.). Partindo disso, os ODS relacionam-se a esse tripé, apontando 17 objetivos, sendo eles: 1º Erradicar a pobreza; 2º Erradicar a fome; 3º Saúde de qualidade; 4º Educação de qualidade; 5º Igualdade de gênero; 6º Água potável e saneamento; 7º Energias renováveis e acessíveis; 8º Trabalho digno e crescimento econômico; 9º Industrial, inovação e infraestruturas; 10º Reduzir as desigualdades; 11º Cidades e comunidades sustentáveis; 12º Consumo e produção responsáveis; 13º Ação contra a mudança global do clima; 14º Vida na água; 15º Vida terrestre; 16º Paz, justiça e instituições eficazes; 17º Parcerias e meios de implementação. Logo, os 17 objetivos podem ser entendidos a partir de cinco esferas de relevância para o planeta e humanidade: a paz; o planeta; as pessoas; a prosperidade; e as parcerias. A soma desses objetivos compõe a chamada Agenda 2030^(7)^.

Salienta-se que o alcance de um objetivo pode ser favorável para o alcance de outros demais. O ODS 5, que diz respeito à igualdade de gênero, por exemplo, influencia diretamente os demais que tratam sobre paz e justiça, segurança alimentar, energia e cidades sustentáveis. Proporcionalmente, criar políticas que permitam a igualdade de gênero dará às mulheres mais visibilidade na sociedade e na economia, permitindo que tenham mais subsídios para lidar com as crises climáticas e participar da criação de políticas direcionadas ao clima^(1)^.

Vale salientar que a desigualdade de gênero faz com que as mulheres recebam os menores salários, componham os altíssimos índices de violência doméstica, feminicídio, bem como os de agressões e assédio sexual, taxas de analfabetismo e pobreza. Este contexto, que vai contra os direitos humanos, torna ainda mais urgente a necessidade de atingir as metas do ODS 5^(1)^.

As problemáticas que se relacionam à questão de gênero são diversas, unindo-se a diferentes camadas do capitalismo e do patriarcado. Reflete-se que, em vários contextos, as mulheres não são introduzidas em aprendizados de habilidades, como nadar ou escalar arvores, que são importantes para as sobrevivências em desastres climáticos, e esses tipos de habilidades são geralmente destinadas a meninos, enquanto as meninas envolvem-se em atividades mais direcionadas ao lar. Durante os desastres, muitas mulheres também não se destinam aos abrigos pelo medo de se afastar dos filhos e de bens domésticos. Além disso, as mulheres também podem sofrer com a falta de acesso a informações confiáveis sobre desastres naturais, que às vezes são transmitidas aos homens em ambientes públicos. Continuadamente, em algumas comunidades, as mulheres também são prejudicadas durante as catástrofes por suas vestimentas restritivas que afetam sua mobilidade na hora de buscar socorro. As mulheres também dispõem de menos aparatos tecnológicos nas buscas por recursos de sobrevivência. Por isso, é tão importante o enfrentamento das desigualdades de gênero^(2)^.

Dentro desse contexto de desigualdade com o gênero feminino, há ainda especificidades que transcendem recortes geográficos, raciais, étnicos e grupais que exigem a criação de metas ainda mais características para essas mulheres. Em países como Brasil, mulheres quilombolas, negras, indígenas, ciganas e quem compõem a comunidade LGBTQI+ estão entre as mais vulneráveis e silenciadas^(6)^.

As mulheres são vistas como protagonistas no alcance dos ODS devido ao fato de que são figuras importantes na busca da cultura da paz, devido à presença de valores, como solidariedade e tolerância, que este público demonstra ao levantar pautas com caráter humanitário. A paz é um caminho importante para o alcance do desenvolvimento sustentável. Assim, a constante premissa pelo fim da violência que as mulheres buscam serve de degrau para o alcance da sustentabilidade e diminuição das mudanças climáticas^(6)^.

Enfatizando-se a relação entre mulheres e clima, perpassando também a discussão de gênero, ao focar no que o ODS 13 defende, as mulheres destacam-se na luta climática pelos seus papéis de promotoras, executoras, criadoras e gestoras em inúmeros contextos sociais, estejam elas os ocupando por vontade própria ou obrigadas a assumir por fatores externos. Geralmente, as mulheres são as figuras responsáveis pela chefia da família, pela organização da casa, pela educação de seus familiares, pela aquisição e preparo de água e alimentos, e pela manutenção familiar, fora suas funções que estão externas ao contexto do lar^(6)^.

Para que as mulheres possam ser mais atuantes no alcance das metas dos ODS, é necessário que haja confluência de ações públicas e da sociedade voltadas para pautas específicas da agenda. Nesse sentido, disputas orçamentárias entre governos, autores e interesses precisam ser vencidas para que a Agenda 30 seja cumprida e as mulheres façam parte desse alcance^(8)^.

## CONSIDERAÇÕES FINAIS

Diante do exposto, compreende-se que o desafio das mudanças climáticas e a busca pelo desenvolvimento sustentável são intrinsecamente ligados à promoção da igualdade de gênero. As mulheres desempenham um papel vital na gestão dos recursos naturais e na resiliência comunitária, mas enfrentam desafios significativos devido às desigualdades de gênero. Para alcançar um futuro sustentável, é imprescindível que as políticas climáticas e de desenvolvimento incluam a participação das mulheres desde a sua fase de concepção. É importante que esses momentos de construção sirvam para acolher e interpretar as capacidades e necessidades das mulheres, garantindo sua plena participação na criação, continuidade e liderança em espaços de governança, assistência e vivência. Somente através de uma abordagem integrada, que une a ação climática com a equidade de gênero, poderemos avançar rumo a um mundo mais justo e sustentável.
